# 4,4′-(Acridine-2,7-di­yl)bis­(2-methyl­but-3-yn-2-ol)

**DOI:** 10.1107/S2414314625009150

**Published:** 2025-10-31

**Authors:** Masaki Yamamura, Seira Ikuma, Tatsuya Nabeshima

**Affiliations:** a Center for Liberal Arts and Science Faculty of Engineering Toyama Prefectural University, 5180 Kurokawa Imizu Toyama 939-0398 Japan; b Graduate School of Pure and Applied Sciences, Tsukuba Research Center for, Interdisciplinary Materials Science (TIMS), University of Tsukuba, 1-1-1, Tennodai, Tsukuba, Ibaraki 305-8571, Japan; University of Antofagasta, Chile

**Keywords:** acridine, π–π stacking, hydrogen bond, crystal structure

## Abstract

The title acridine derivative, C_23_H_21_NO_2_, which has two 2-methyl­but-3-yn-2-ol moieties at the 2,7-positions, was synthesized by Sonogashira coupling reaction. In the crystal, a columnar structure is formed by the π–π stacking of acridine units. The 2-methyl­but-3-yn-2-ol moieties form inter­molecular hydrogen bonds.

## Abstract 2

The title acridine derivative, which has two 2-methyl­but-3-yn-2-ol moieties at the 2,7-positions, was synthesized by Sonogashira coupling reaction. In crystal, a columnar structure is formed by the π–π stacking of acridine units. The 2-methyl­but-3-yn-2-ol moieties form inter­molecular hydrogen bonds.

## Graphical abstract

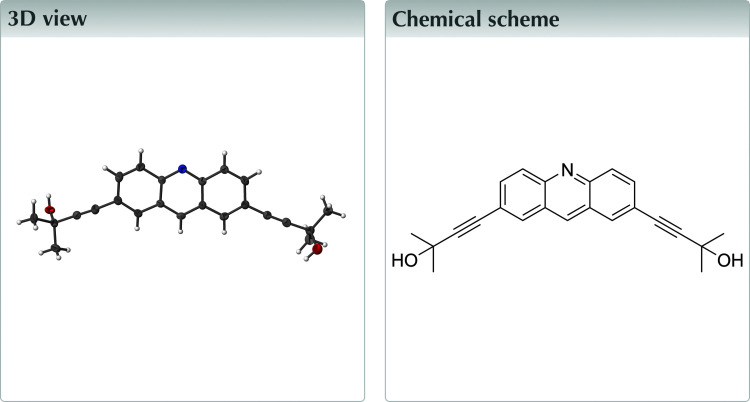

## Structure description

Acridine is often used as a luminophore (Ryan *et al.*, 1997 ▸) and DNA-inter­calator (Lerman, 1963 ▸). It is known to crystallize in seven polymorphic forms, in which various inter­molecular inter­actions, *i.e.*, π–π, C—H–π, and C—H–N inter­actions, are observed (Mei & Wolf, 2004 ▸).

**Figure 1:**
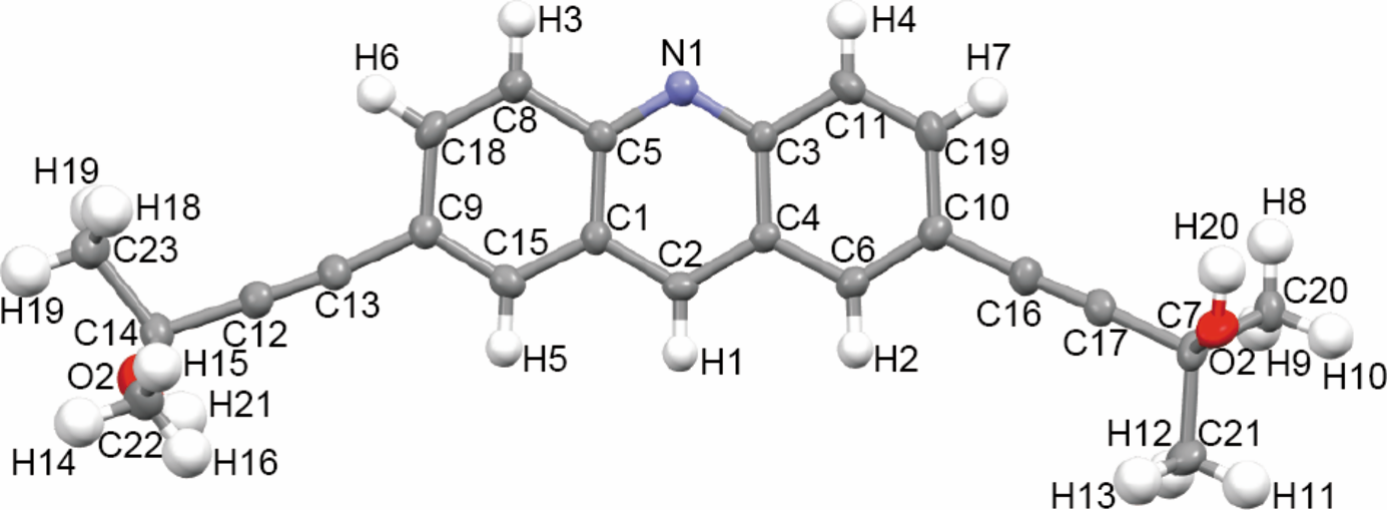
Mol­ecular structure of the title compound with 50% probability ellipsoids.

**Figure 2:**
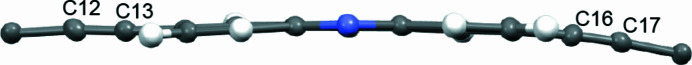
Side view of mol­ecular structure. Terminal methyl and hy­droxy groups are omitted for clarify.

The title compound (Fig. 1 ▸) was synthesized by the Sonogashira coupling reaction of 2,7-di­bromo­acridine with 2-methyl­but-3-yn-2-ol. The structure of the core acridine unit of the title compound is very similar to those of other 2,7-substituted acridine (Yamamura *et al.*, 2015 ▸). All the bond lengths and angles in the acridine unit are in expected ranges. The C12 and C13 atoms in a triple bond are within the least-square plane of the acridine unit (Fig. 2 ▸). In contrast, the C16 and C17 atoms in the other triple bond are separated from the plane. The distance of C16 from the plane is 0.179 (3) Å and that of C17 is 0.331 (3) Å.

**Figure 3:**
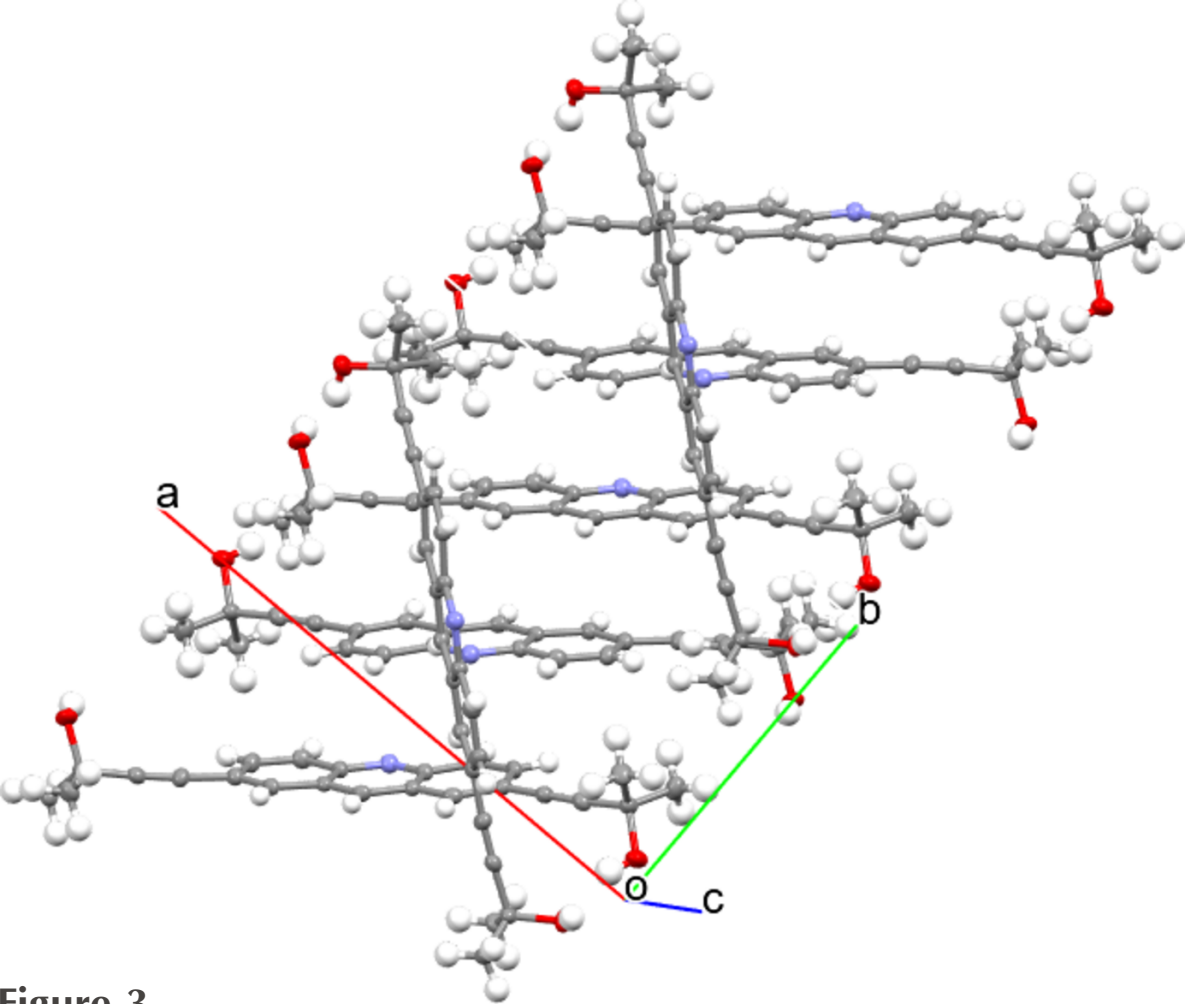
Packing of the title compound

In the crystal, a columnar structure was observed due to the π–π stacking of acridine units (Fig. 3 ▸). The distance between the least-square planes of the acridine units is 3.505  Å. The acridine unit is arranged in anti-fashion toward a neighbor acridine unit. Inter­molecular hydrogen bonds also link mol­ecules (Table 1 ▸). Two hy­droxy groups of **1** form hydrogen bonds with two different mol­ecules, between which another mol­ecule is inserted.

## Synthesis and crystallization

A mixture of 2,7-di­bromo­acridine (Vlassa *et al.*, 1995 ▸) (0.158 g, 47.0 mmol), 2-methyl­but-3-yn-2-ol (0.15 ml, 1.5 mmol), PdCl_2_(PPh_3_)_2_ (6.7 mg, 2 mol%), and CuI (1.6 mg, 2 mol%) were refluxed for 15 h in diiso­propyl­amine (45 ml). After evaporation, the residue was extracted with CH_2_Cl_2_ then washed with water. After evaporation, the crude products were separated by silica-gel column chromatography to give the yellow powder of the title compound in 43% yield. Yellow crystals suitable for X-ray analysis were obtained form a CHCl_3_/hexane solution.

^1^H NMR (300 MHz, CDCl_3_): δ 8.62 (*s*, 1H), 8.14 (*d*, *J* = 8.9 Hz, 2H), 8.09 (*d*, *J* = 1.7 Hz, 2H), 7.73 (*dd*, *J* = 1.7, 8.9 Hz, 2H), 2.08 (*s*, 2H), 1.70 (*s*, 12H); ^13^C NMR (100 MHz, CDCl_3_): δ 149.1 (CH), 136.2 (C), 133.9 (CH), 132.3 (CH), 130.2 (CH), 127.1 (CH), 121.3 (C), 84.7 (C), 82.6 (C), 66.3 (C), 31.7 (CH_3_); Analysis calculated for C_23_H_21_NO_2_: C, 80.44; H, 6.16; N, 4.08; Found: C, 80.13; H, 5.99; N, 3.89.

## Refinement

Crystal data, data collection and structure refinement details are summarized in Table 2 ▸.

## Supplementary Material

Crystal structure: contains datablock(s) I. DOI: 10.1107/S2414314625009150/bx4038sup1.cif

Structure factors: contains datablock(s) I. DOI: 10.1107/S2414314625009150/bx4038Isup2.hkl

Supporting information file. DOI: 10.1107/S2414314625009150/bx4038Isup3.cml

CCDC reference: 2496569

Additional supporting information:  crystallographic information; 3D view; checkCIF report

## Figures and Tables

**Table 1:** Hydrogen-bond geometry (Å, °)

*D*—H⋯*A*	*D*—H	H⋯*A*	*D*⋯*A*	*D*—H⋯*A*
O1—H20⋯O2^i^	0.84	2.04	2.794 (4)	149
O2—H21⋯O1^ii^	0.84	1.94	2.735 (4)	158

Symmetry codes: (i) 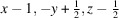; (ii) 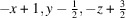.

**Table 2:** Experimental details

Crystal data	
Chemical formula	C_23_H_21_NO_2_
*M*_r_	343.41
Crystal system, space group	Monoclinic, *P*2_1_/*c*
Temperature (K)	120
*a*, *b*, *c* (Å)	16.023 (13), 9.496 (7), 12.215 (10)
β (°)	99.646 (10)
*V* (Å^3^)	1832 (2)
*Z*	4
Radiation type	Mo *K*α
μ (mm^−1^)	0.08
Crystal size (mm)	0.20 × 0.20 × 0.05

Data collection	
Diffractometer	Bruler APEXII CCD
Absorption correction	Multi-scan (*SADABS*; Krause *et al.*, 2015 ▸)
*T*_min_, *T*_max_	0.843, 0.915
No. of measured, independent and observed [*I* > 2σ(*I*)] reflections	10340, 3058, 1605
*R*_int_	0.093
(sin θ/λ)_max_ (Å^−1^)	0.585

Refinement	
*R*[*F*^2^ > 2σ(*F*^2^)], *wR*(*F*^2^), *S*	0.056, 0.122, 1.01
No. of reflections	3058
No. of parameters	241
H-atom treatment	H-atom parameters constrained
Δρ_max_, Δρ_min_ (e Å^−3^)	0.32, −0.28

Computer programs: *APEX2* and *SAINT* (Bruker, 2014 ▸), *SHELXT* (Sheldrick, 2015*a* ▸) , *SHELXL2019*/1 (Sheldrick, 2015*b* ▸) and *Mercury* (Macrae *et al.*, 2020 ▸).

## full crystallographic data

### 4,4'-(Acridine-2,7-diyl)bis(2-methylbut-3-yn-2-ol) Crystal data

**Table:**

C_23_H_21_NO_2_	*F*(000) = 728
*M**_r_* = 343.41	*D*_x_ = 1.245 Mg m^−^^3^
Monoclinic, *P*2_1_/*c*	Mo *K*α radiation, λ = 0.71073 Å
*a* = 16.023 (13) Å	Cell parameters from 476 reflections
*b* = 9.496 (7) Å	θ = 2.5–26.6°
*c* = 12.215 (10) Å	µ = 0.08 mm^−^^1^
β = 99.646 (10)°	*T* = 120 K
*V* = 1832 (2) Å^3^	Prism, colorless
*Z* = 4	0.20 × 0.20 × 0.05 mm

### 4,4'-(Acridine-2,7-diyl)bis(2-methylbut-3-yn-2-ol) Data collection

**Table:**

Bruler APEXII CCD diffractometer	1605 reflections with *I* > 2σ(*I*)
Radiation source: fine-focus seald tube	*R*_int_ = 0.093
φ and ω scan	θ_max_ = 24.6°, θ_min_ = 1.3°
Absorption correction: multi-scan (SADABS; Krause *et al.*, 2015)	*h* = −18→18
*T*_min_ = 0.843, *T*_max_ = 0.915	*k* = −11→11
10340 measured reflections	*l* = −14→11
3058 independent reflections	

### 4,4'-(Acridine-2,7-diyl)bis(2-methylbut-3-yn-2-ol) Refinement

**Table:**

Refinement on *F*^2^	0 restraints
Least-squares matrix: full	Hydrogen site location: inferred from neighbouring sites
*R*[*F*^2^ > 2σ(*F*^2^)] = 0.056	H-atom parameters constrained
*wR*(*F*^2^) = 0.122	*w* = 1/[σ^2^(*F*_o_^2^) + (0.030*P*)^2^ + 1.*P*] where *P* = (*F*_o_^2^ + 2*F*_c_^2^)/3
*S* = 1.01	(Δ/σ)_max_ < 0.001
3058 reflections	Δρ_max_ = 0.32 e Å^−^^3^
241 parameters	Δρ_min_ = −0.28 e Å^−^^3^

### 4,4'-(Acridine-2,7-diyl)bis(2-methylbut-3-yn-2-ol) Special details

**Table:**

Geometry. All esds (except the esd in the dihedral angle between two l.s. planes) are estimated using the full covariance matrix. The cell esds are taken into account individually in the estimation of esds in distances, angles and torsion angles; correlations between esds in cell parameters are only used when they are defined by crystal symmetry. An approximate (isotropic) treatment of cell esds is used for estimating esds involving l.s. planes.

### 4,4'-(Acridine-2,7-diyl)bis(2-methylbut-3-yn-2-ol) Fractional atomic coordinates and isotropic or equivalent isotropic displacement parameters (Å^2^)

**Table:**

	*x*	*y*	*z*	*U*_iso_*/*U*_eq_	
O1	0.05976 (13)	0.6608 (2)	0.54622 (18)	0.0336 (6)	
H20	0.046205	0.632400	0.480520	0.050*	
O2	0.95459 (13)	−0.0895 (2)	0.8478 (2)	0.0391 (7)	
H21	0.936554	−0.016649	0.875071	0.059*	
N1	0.49494 (16)	0.2007 (3)	0.3889 (2)	0.0259 (7)	
C1	0.57022 (19)	0.1892 (3)	0.5807 (3)	0.0222 (8)	
C2	0.51585 (19)	0.2890 (3)	0.6130 (3)	0.0236 (8)	
H1	0.523062	0.319641	0.688064	0.028*	
C3	0.44299 (19)	0.2968 (3)	0.4223 (3)	0.0221 (8)	
C4	0.45037 (19)	0.3441 (3)	0.5345 (3)	0.0201 (8)	
C5	0.55663 (19)	0.1465 (3)	0.4664 (3)	0.0227 (8)	
C6	0.39012 (19)	0.4417 (3)	0.5630 (3)	0.0238 (8)	
H2	0.394231	0.472234	0.637781	0.029*	
C7	0.1346 (2)	0.7473 (3)	0.5551 (3)	0.0269 (8)	
C8	0.60864 (19)	0.0387 (3)	0.4338 (3)	0.0246 (8)	
H3	0.600074	0.008741	0.358542	0.030*	
C9	0.68701 (19)	0.0220 (3)	0.6217 (3)	0.0246 (8)	
C10	0.3259 (2)	0.4925 (3)	0.4836 (3)	0.0263 (9)	
C11	0.3759 (2)	0.3515 (3)	0.3423 (3)	0.0275 (9)	
H4	0.370215	0.321267	0.267231	0.033*	
C12	0.8143 (2)	−0.1025 (3)	0.7458 (3)	0.0286 (9)	
C13	0.7548 (2)	−0.0444 (3)	0.6939 (3)	0.0257 (8)	
C14	0.88472 (19)	−0.1841 (3)	0.8114 (3)	0.0277 (8)	
C15	0.63762 (19)	0.1265 (3)	0.6553 (3)	0.0251 (8)	
H5	0.648782	0.157535	0.730252	0.030*	
C16	0.2620 (2)	0.5886 (3)	0.5099 (3)	0.0274 (9)	
C17	0.2065 (2)	0.6609 (3)	0.5275 (3)	0.0256 (8)	
C18	0.6699 (2)	−0.0219 (3)	0.5076 (3)	0.0274 (9)	
H6	0.702629	−0.095674	0.483679	0.033*	
C19	0.3198 (2)	0.4463 (3)	0.3715 (3)	0.0293 (9)	
H7	0.276038	0.482228	0.316502	0.035*	
C20	0.1141 (2)	0.8677 (3)	0.4733 (3)	0.0307 (9)	
H8	0.102475	0.830157	0.397582	0.046*	
H9	0.162315	0.932392	0.480441	0.046*	
H10	0.064129	0.918263	0.489222	0.046*	
C21	0.1557 (2)	0.7998 (3)	0.6747 (3)	0.0335 (9)	
H11	0.108256	0.855329	0.692606	0.050*	
H12	0.206642	0.858645	0.683083	0.050*	
H13	0.165849	0.719074	0.725186	0.050*	
C22	0.8534 (2)	−0.2498 (3)	0.9101 (3)	0.0352 (9)	
H14	0.899577	−0.302739	0.954676	0.053*	
H15	0.806286	−0.313714	0.883803	0.053*	
H16	0.834173	−0.175585	0.955753	0.053*	
C23	0.9165 (2)	−0.2961 (3)	0.7380 (3)	0.0382 (10)	
H17	0.941707	−0.249937	0.679546	0.057*	
H18	0.868982	−0.355098	0.704005	0.057*	
H19	0.959236	−0.354900	0.783358	0.057*	

### 4,4'-(Acridine-2,7-diyl)bis(2-methylbut-3-yn-2-ol) Atomic displacement parameters (Å^2^)

**Table:**

	*U*^11^	*U*^22^	*U*^33^	*U*^12^	*U*^13^	*U*^23^
O1	0.0267 (13)	0.0324 (14)	0.0409 (18)	−0.0101 (11)	0.0031 (12)	−0.0138 (12)
O2	0.0330 (15)	0.0263 (14)	0.0553 (19)	−0.0053 (12)	−0.0010 (13)	−0.0019 (13)
N1	0.0270 (16)	0.0263 (15)	0.0247 (19)	0.0009 (14)	0.0048 (14)	0.0022 (13)
C1	0.0202 (18)	0.0209 (17)	0.026 (2)	−0.0004 (15)	0.0052 (16)	0.0019 (16)
C2	0.0246 (19)	0.0260 (18)	0.020 (2)	−0.0046 (16)	0.0036 (16)	−0.0035 (16)
C3	0.0223 (19)	0.0203 (18)	0.024 (2)	0.0011 (15)	0.0054 (16)	0.0012 (16)
C4	0.0214 (18)	0.0194 (17)	0.021 (2)	−0.0019 (15)	0.0069 (16)	−0.0002 (15)
C5	0.0240 (19)	0.0218 (18)	0.023 (2)	−0.0006 (16)	0.0048 (17)	0.0032 (16)
C6	0.025 (2)	0.0231 (18)	0.025 (2)	−0.0008 (16)	0.0082 (17)	−0.0020 (16)
C7	0.0214 (19)	0.0197 (17)	0.039 (2)	−0.0002 (15)	0.0037 (16)	−0.0033 (16)
C8	0.025 (2)	0.0248 (18)	0.025 (2)	0.0012 (16)	0.0070 (17)	0.0004 (16)
C9	0.0196 (19)	0.0246 (19)	0.030 (3)	−0.0018 (16)	0.0048 (17)	0.0004 (17)
C10	0.025 (2)	0.0237 (18)	0.031 (3)	0.0000 (16)	0.0081 (18)	0.0005 (17)
C11	0.030 (2)	0.0279 (19)	0.023 (2)	−0.0008 (17)	−0.0002 (17)	0.0030 (16)
C12	0.033 (2)	0.0233 (18)	0.031 (2)	0.0005 (17)	0.0073 (18)	0.0015 (17)
C13	0.025 (2)	0.0222 (18)	0.030 (2)	−0.0017 (17)	0.0034 (17)	−0.0004 (16)
C14	0.0227 (19)	0.0238 (18)	0.035 (2)	−0.0020 (16)	0.0001 (16)	−0.0005 (17)
C15	0.026 (2)	0.0258 (19)	0.023 (2)	−0.0051 (16)	0.0016 (16)	0.0018 (16)
C16	0.023 (2)	0.0285 (19)	0.032 (2)	0.0013 (17)	0.0071 (17)	0.0038 (17)
C17	0.027 (2)	0.0202 (18)	0.029 (2)	−0.0016 (16)	0.0020 (16)	0.0013 (15)
C18	0.026 (2)	0.0225 (19)	0.036 (3)	0.0017 (16)	0.0114 (18)	−0.0033 (17)
C19	0.024 (2)	0.030 (2)	0.034 (3)	0.0030 (16)	0.0052 (17)	0.0085 (18)
C20	0.0251 (19)	0.0263 (19)	0.040 (2)	0.0039 (15)	0.0053 (17)	0.0047 (17)
C21	0.039 (2)	0.0278 (19)	0.034 (2)	−0.0010 (17)	0.0065 (18)	−0.0082 (17)
C22	0.040 (2)	0.035 (2)	0.032 (2)	0.0013 (18)	0.0117 (18)	0.0105 (18)
C23	0.029 (2)	0.045 (2)	0.040 (3)	0.0041 (18)	0.0023 (18)	−0.0062 (19)

### 4,4'-(Acridine-2,7-diyl)bis(2-methylbut-3-yn-2-ol) Geometric parameters (Å, º)

**Table:**

O1—C7	1.442 (4)	C10—C19	1.425 (5)
O1—H20	0.8400	C10—C16	1.448 (4)
O2—C14	1.446 (4)	C11—C19	1.362 (4)
O2—H21	0.8400	C11—H4	0.9500
N1—C3	1.344 (4)	C12—C13	1.189 (4)
N1—C5	1.351 (4)	C12—C14	1.487 (5)
C1—C2	1.388 (4)	C14—C22	1.516 (4)
C1—C15	1.422 (4)	C14—C23	1.533 (4)
C1—C5	1.435 (4)	C15—H5	0.9500
C2—C4	1.400 (4)	C16—C17	1.171 (4)
C2—H1	0.9500	C18—H6	0.9500
C3—C11	1.424 (4)	C19—H7	0.9500
C3—C4	1.427 (4)	C20—H8	0.9800
C4—C6	1.423 (4)	C20—H9	0.9800
C5—C8	1.419 (4)	C20—H10	0.9800
C6—C10	1.377 (4)	C21—H11	0.9800
C6—H2	0.9500	C21—H12	0.9800
C7—C17	1.498 (4)	C21—H13	0.9800
C7—C20	1.518 (4)	C22—H14	0.9800
C7—C21	1.527 (4)	C22—H15	0.9800
C8—C18	1.346 (4)	C22—H16	0.9800
C8—H3	0.9500	C23—H17	0.9800
C9—C15	1.374 (4)	C23—H18	0.9800
C9—C13	1.427 (4)	C23—H19	0.9800
C9—C18	1.436 (4)		
			
C7—O1—H20	109.5	O2—C14—C12	108.7 (3)
C14—O2—H21	109.5	O2—C14—C22	110.7 (3)
C3—N1—C5	117.6 (3)	C12—C14—C22	108.7 (3)
C2—C1—C15	123.3 (3)	O2—C14—C23	107.1 (2)
C2—C1—C5	118.0 (3)	C12—C14—C23	110.0 (3)
C15—C1—C5	118.7 (3)	C22—C14—C23	111.5 (3)
C1—C2—C4	119.6 (3)	C9—C15—C1	121.6 (3)
C1—C2—H1	120.2	C9—C15—H5	119.2
C4—C2—H1	120.2	C1—C15—H5	119.2
N1—C3—C11	118.2 (3)	C17—C16—C10	175.8 (4)
N1—C3—C4	123.3 (3)	C16—C17—C7	176.7 (3)
C11—C3—C4	118.4 (3)	C8—C18—C9	121.7 (3)
C2—C4—C6	122.5 (3)	C8—C18—H6	119.2
C2—C4—C3	118.2 (3)	C9—C18—H6	119.2
C6—C4—C3	119.3 (3)	C11—C19—C10	120.9 (3)
N1—C5—C8	118.3 (3)	C11—C19—H7	119.5
N1—C5—C1	123.3 (3)	C10—C19—H7	119.5
C8—C5—C1	118.4 (3)	C7—C20—H8	109.5
C10—C6—C4	120.9 (3)	C7—C20—H9	109.5
C10—C6—H2	119.6	H8—C20—H9	109.5
C4—C6—H2	119.6	C7—C20—H10	109.5
O1—C7—C17	109.4 (2)	H8—C20—H10	109.5
O1—C7—C20	107.1 (3)	H9—C20—H10	109.5
C17—C7—C20	110.8 (3)	C7—C21—H11	109.5
O1—C7—C21	107.8 (3)	C7—C21—H12	109.5
C17—C7—C21	109.8 (3)	H11—C21—H12	109.5
C20—C7—C21	111.9 (3)	C7—C21—H13	109.5
C18—C8—C5	121.2 (3)	H11—C21—H13	109.5
C18—C8—H3	119.4	H12—C21—H13	109.5
C5—C8—H3	119.4	C14—C22—H14	109.5
C15—C9—C13	123.5 (3)	C14—C22—H15	109.5
C15—C9—C18	118.3 (3)	H14—C22—H15	109.5
C13—C9—C18	118.3 (3)	C14—C22—H16	109.5
C6—C10—C19	119.4 (3)	H14—C22—H16	109.5
C6—C10—C16	122.5 (3)	H15—C22—H16	109.5
C19—C10—C16	118.0 (3)	C14—C23—H17	109.5
C19—C11—C3	121.0 (3)	C14—C23—H18	109.5
C19—C11—H4	119.5	H17—C23—H18	109.5
C3—C11—H4	119.5	C14—C23—H19	109.5
C13—C12—C14	175.9 (3)	H17—C23—H19	109.5
C12—C13—C9	174.1 (4)	H18—C23—H19	109.5
			
C15—C1—C2—C4	−179.2 (3)	C3—C4—C6—C10	−1.1 (4)
C5—C1—C2—C4	0.0 (4)	N1—C5—C8—C18	−178.4 (3)
C5—N1—C3—C11	−178.0 (3)	C1—C5—C8—C18	−0.5 (4)
C5—N1—C3—C4	0.4 (4)	C4—C6—C10—C19	0.1 (4)
C1—C2—C4—C6	177.0 (3)	C4—C6—C10—C16	178.1 (3)
C1—C2—C4—C3	−1.1 (4)	N1—C3—C11—C19	178.3 (3)
N1—C3—C4—C2	1.0 (4)	C4—C3—C11—C19	−0.2 (4)
C11—C3—C4—C2	179.3 (3)	C13—C9—C15—C1	−179.6 (3)
N1—C3—C4—C6	−177.3 (3)	C18—C9—C15—C1	0.9 (4)
C11—C3—C4—C6	1.1 (4)	C2—C1—C15—C9	176.1 (3)
C3—N1—C5—C8	176.1 (3)	C5—C1—C15—C9	−3.1 (4)
C3—N1—C5—C1	−1.6 (4)	C5—C8—C18—C9	−1.7 (5)
C2—C1—C5—N1	1.4 (4)	C15—C9—C18—C8	1.6 (5)
C15—C1—C5—N1	−179.3 (3)	C13—C9—C18—C8	−178.0 (3)
C2—C1—C5—C8	−176.3 (3)	C3—C11—C19—C10	−0.8 (5)
C15—C1—C5—C8	2.9 (4)	C6—C10—C19—C11	0.8 (5)
C2—C4—C6—C10	−179.2 (3)	C16—C10—C19—C11	−177.3 (3)

### 4,4'-(Acridine-2,7-diyl)bis(2-methylbut-3-yn-2-ol) Hydrogen-bond geometry (Å, º)

**Table:**

*D*—H···*A*	*D*—H	H···*A*	*D*···*A*	*D*—H···*A*
O1—H20···O2^i^	0.84	2.04	2.794 (4)	149
O2—H21···O1^ii^	0.84	1.94	2.735 (4)	158

Symmetry codes: (i) *x*−1, −*y*+1/2, *z*−1/2; (ii) −*x*+1, *y*−1/2, −*z*+3/2.
